# Comorbidity, Treatment, and Service Utilization Patterns in Difficult-to-Treat Depression Patients: A Retrospective Study in a Portuguese Community Mental Health Team

**DOI:** 10.3390/medicina60111734

**Published:** 2024-10-22

**Authors:** João Gouveia, Marta Moura Neves, Nuno Madeira, Vítor Santos, António Macedo

**Affiliations:** 1 Faculty of Medicine, University of Coimbra (UC), 3004-531 Coimbra, Portugal; joaoasdgouveia@outlook.com (J.G.);; 2 Department of Psychiatry, Unidade Local de Saúde de Coimbra (ULS-C), 3004-561 Coimbra, Portugal; 3 Coimbra Institute for Biomedical Imaging and Translational Research (CIBIT), Institute of Nuclear Sciences Applied to Health (ICNAS), University of Coimbra (UC), 3000-548 Coimbra, Portugal

**Keywords:** difficult-to-treat depression, Portuguese community mental health team, real-world data

## Abstract

*Background and Objectives*: Observational studies with data from real-world clinical practice with patients with difficult-to-treat depression (DTD) are rare. This study aims to collect observational data from the real-world clinical practice of a Portuguese community mental health team (CMHT) on the prevalence of DTD and to explore differences between DTD and non-DTD groups. *Materials and Methods*: We conducted a retrospective chart review study using data from Electronic Health Records (EHRs) of adult patients with psychiatric disorders followed by a CMHT from the Department of Psychiatry of the Coimbra Local Health Unit (between 1 December 2020–31 December 2022). The Dutch Measure for quantification of Treatment Resistance in Depression (DM-TRD) was used to assess the degree of treatment resistance and the Charlson Comorbidity Index (CCI) to measure medical comorbidity. *Results*: A quantity of 473 patients were referred to Cantanhede CMHT for a first assessment. Of these, 219 patients met the criteria for a primary diagnosis of any depressive disorder. Assistant psychiatrists identified 57 patients with DTD during follow-up (approximately 26%). The DTD group had higher rates of depressive episodes, greater depression severity, increased service use, higher DM-TRD scores, and a higher prevalence of comorbid anxiety symptoms, personality disorders, and severe medical comorbidities. The DTD group also had a higher prescription rate of antidepressants. Differences were observed in the use of antidepressant augmentation strategies and in the prescription of anticoagulant/antiplatelet drugs and analgesics, with higher prescription rates in the DTD group. We found correlations between DM-TRD and CCI scores, and between DM-TRD scores and all service use variables. *Conclusions*: Our results are consistent with a similar study in the United Kingdom, highlighting the need for a different approach to the management of DTD patients, who continue to live with a significant burden despite usual pharmacological and non-pharmacological treatments.

## 1. Introduction

Depressive disorders are one of the leading causes of the global burden of disease, affecting millions of people worldwide. They cause considerable suffering to patients and their families, impair social functioning and economic productivity, and are associated with premature mortality from suicide and medical comorbidities, leading to a substantial demand for mental health services [1]. Treatment of depressive disorders commonly involves pharmacological therapy with antidepressant medications, psychotherapy, or a combination of both [2]. Lifestyle interventions (e.g., exercise) and neuromodulation techniques (e.g., electroconvulsive therapy, transcranial magnetic stimulation) are also therapeutic options with robust evidence of effectiveness in depression [3,4].

Despite the availability of various treatment modalities, a significant proportion of patients with depression fail to achieve remission or even significant symptom improvement. Almost a third of patients with a depressive episode will not achieve sustained remission, even with several well-delivered treatments prescribed by their doctor. These patients experience prolonged suffering and are heavy consumers of mental health care services, resulting in high costs for health resources for years [5]. Furthermore, relapse and recurrence of depression are common, adding to the overall burden of depression [6]. These phenomena have spurred the delineation of two distinct but related concepts within the realm of depression treatment refractoriness: treatment-resistant depression (TRD) and difficult-to-treat depression (DTD).

It is currently estimated that at least 30% of people with depression could have TRD, and various definitions have been proposed, associated with different conceptual frameworks [7,8]. The definition of TRD adopted by regulatory agencies such as the United States Food and Drug Administration (FDA) and the European Medicines Agency (EMA) consists of response failure to two or more antidepressant regimens, despite adequate dose, duration, and adherence to treatment [9,10]. Other authors have proposed a staging model that operationalizes TRD in a dimensional continuum of failed antidepressant trials [11]. However, these definitions have some limitations, such as the operationalization of “failure” of treatment, the definition of clinical characteristics of depression (e.g., severity, duration), and the failure to include other treatment modalities besides pharmacological ones [8]. To address some of these limitations, the Maudsley Staging Model (MSM) was developed. It defines treatment resistance as the failure to attain a significant level of improvement from an accurately diagnosed depressive episode following treatment with an antidepressant given at an adequate dose for a minimum of six weeks [12]. Three dimensions of resistance are included in this model: treatment failure, duration of the depressive episode, and severity of depression. The Dutch Measure for quantification of Treatment-Resistant Depression Model (DM-TRD) is the most comprehensive in terms of variables included and was developed to improve the MSM [13]. Items for functional impairment, comorbid anxiety, personality disorders, psychosocial stressors, failed psychotherapy, and intensified treatment were added, and it was shown to have better predictive validity for clinical outcomes than previous models used in the quantification of TRD, resulting in improved treatment for TRD patients [13].

The DTD construct represents a broader and more inclusive approach to the outcome of depression treatment, encompassing psychological, biological, and interactive aspects for an integrative model of therapeutic management of resistance in depression [14]. An international consensus, comprising 15 clinical researchers from Europe, the United States, Canada, and Australia with expertise in mood disorders, was established in 2020 and defined DTD as depression that continues to cause a significant burden despite normal treatment efforts [15]. This expert panel conceptualized DTD as a dimensional construct, without explicitly specifying criteria to distinguish patients who did and did not have DTD. However, this clinical phenomenon lies on a spectrum that includes partial response, nonresponse, and frequent relapses, shifting the treatment focus from an acute objective of remission to a disease management model that emphasizes symptom control, better functioning and quality of life, and minimization of therapy side effects [15,16]. The consensus statement identified multiple variables associated with DTD and emphasized the importance of conducting a comprehensive evaluation to identify possible contributors to inadequate treatment response. Some of the patient and disorder characteristics that predict poorer outcomes in the treatment of depression include prior nonresponse to treatment, symptom chronicity, personality pathology, comorbid disorders, childhood trauma, suicidality, substance misuse, psychosocial stress, social isolation, and early age of onset [15]. Recently, a self-report scale that can be incorporated into clinical practice to identify patient, clinical, and treatment risk factors for DTD was developed [17].

There are few studies about TRD in Portugal. One epidemiological study estimated the prevalence of TRD and quantified the disease burden (disability-adjusted life year—DALY) due to the disability generated by TRD in Portugal in 2017, based on data from the National Epidemiological Study of Mental Health. The estimated prevalence of TRD was 90.7 thousand persons, and the estimated disease burden due to the disability generated by TRD was 25.7 thousand disability-adjusted life years (DALYs). The authors also concluded that although TRD represents relatively small direct costs for the health system, it had a significant disease burden and substantial productivity costs for the Portuguese economy and society due to reduced employment, absenteeism, presenteeism, and premature death [18]. Another article focused on the perspective of a panel of seven Portuguese psychiatry experts to characterize and discuss TRD epidemiology, diagnosis, patient care pathways, treatment options, and unmet clinical needs. They reached consensual statements that TRD diagnosis and treatment are mostly decided by psychiatrists at public hospitals; treatment type and duration must be adapted to the characteristics of the patient and the depressive episode; antidepressant switch interventions occur more frequently with non-response, while optimization, combination, and augmentation strategies are considered for patients with a partial response; psychotherapy should be considered in parallel to pharmacological treatment; neuromodulation techniques are underused; lifelong treatment is required for recurrent or more chronic TRD episodes; and TRD management is limited by lack of access to specialist care and many treatment options [19].

Observational studies with data from real-world clinical practice on patients identified with DTD are even scarcer, despite its clinical relevance. To our knowledge, a single study analyzed the electronic health records (EHRs) of five specialist mental health National Health Service (NHS) Trusts in the United Kingdom (UK) using a natural language processing model. Data on disease characteristics, comorbidities, and treatment histories were extracted. In a sample of 28,184 patients with major depressive disorder, 19% met the criteria for DTD. Compared to the non-DTD group, patients with DTD were more likely to have severe depression, suicidal ideation, and psychiatric and medical comorbidities, as well as higher rates of hospitalization. They were also more likely to receive unemployment and sickness/disability benefits. More intensive treatment strategies were used in the DTD group, including higher rates of combination therapy, augmentation, psychotherapy, and electroconvulsive therapy [20]. To our knowledge, there are no other DTD observational studies with data from real-world clinical practice, namely in the Portuguese context.

The objectives of the present study were to gather and analyze observational data from real-world clinical practice of a Portuguese community mental health team (CMHT) about:

(1) The prevalence of DTD in patients with depressive disorders treated by a CMHT.
(2) Differences between the DTD and non-DTD groups in depression treatment resistance; psychiatric and medical comorbidity; use of different antidepressant pharmacological strategies; use of other treatment modalities and service use patterns. We hypothesize that the DTD group, compared with the non-DTD group, will have higher scores in TRD multidimensional measures, higher rates of psychiatric and medical comorbidities, more frequent use of antidepressant combination and augmentation strategies, more frequent use of psychotherapeutic and neuromodulatory interventions, more psychiatric consultations, more psychiatric hospitalization days, and more emergency room visits during the follow-up.
(3) Associations and relations between TRD multidimensional scores, medical comorbidity scores, and use variables.

We hypothesized that distinct disease and treatment characteristics were associated with DTD, namely a higher consumption of service care.

## 2. Materials and Methods

### 2.1. Design and Sample

This retrospective chart review study was conducted in Portugal using data from EHRs of patients followed by a single CMHT from the Department of Psychiatry of Coimbra Hospital University Centre: the Cantanhede CMHT, stablished in January 2020. Cantanhede CMHT is a multidisciplinary mental health team responsible for the specialized care of adults (18 years or older) with psychiatric disorders referred from primary care centers of Cantanhede and Mira (approximately 47,000 inhabitants), in coordination with other psychiatric department units (inpatient units, day care hospital, psychiatric emergency services, specialized ambulatory teams). In order to better replicate usual pathways of care for depression in the outpatient setting, the study population included individuals who had their first consultation with one of the Cantanhede CMHT psychiatrists between the start of its operation, 1 January 2020, and 31 December 2022, with a primary diagnosis of depressive disorder, defined by the International Classification of Diseases, Eleventh Revision (ICD-11), codes 6A7x [21]. Patients with a primary diagnosis of a neurodevelopmental, neurocognitive, primary psychotic, or bipolar disorder were excluded. Patients with DTD were identified by one of the two practicing psychiatrists of the CMHT, based on the broad definition of the international consensus panel (“depression that continues to cause a significant burden despite normal treatment efforts”) and considering factors such as the chronicity of the current episode, the frequency of recurrences, and a history of multiple antidepressant treatments from different modalities (psychopharmacologic, psychotherapeutic, or neuromodulation interventions) [15]. According to the consensus, patients with DTD lack acute phase response/remission or do not sustain the acute response/remission, with significant burden related to impairments in daily function, quality of life, and/or symptoms of treatment side effects. The relevant study data were extracted and analyzed by two authors from the EHRs of all eligible patients and were pseudo-anonymized through the assignment of a numeric code to each patient paired with their respective data. The data obtained were stored in a computerized database, protected by a password.

### 2.2. Data and Measures

Extracted data from EHRs included sociodemographic information (age, gender), psychiatric diagnoses formulated by the CMHT psychiatrist based on the ICD-11, relevant clinical and treatment history data, and medical history to compute scores of depression treatment resistance and medical comorbidity measures [21]. Additionally, data from treatment options during follow-up and service use data (psychiatric consultations, emergency room visits, psychiatric hospitalization days) were collected. DM-TRD [13] was used to grade the treatment resistance level, assigning a score varying between 2 and 27.

The original version of the Charlson Comorbidity Index (CCI) was used to measure medical comorbidity [22]. This measure contains 19 items corresponding to different medical comorbid conditions with different clinical weights based on the adjusted risk of 1-year mortality [22]. The conditions with an assigned weight of 1 are: myocardial infarction, congestive heart failure, peripheral vascular disease, cerebrovascular disease, dementia, chronic pulmonary disease, connective tissue disease, ulcer disease, mild liver disease, and diabetes. The conditions with an assigned weight of 2 are: hemiplegia, moderate or severe renal disease, diabetes with end organ damage, any tumor without metastasis, leukemia, and lymphoma. Moderate or severe liver disease has an assigned weight of 3 and metastatic solid tumor and Acquired Immune Deficiency Syndrome have an assigned weight of 6. The CCI total score consists in a simple sum of the weights, with higher scores indicating a greater mortality risk and a more severe comorbid condition [23].

Data extracted on treatment history included the use of different groups of antidepressants (selective serotonin reuptake inhibitors—SSRI, serotonin noradrenaline reuptake inhibitors—SNRI, bupropiom, mirtazapine, trazodone, agomelatine, vortioxetine, tricyclic antidepressants); use of antidepressant combination and augmentation strategies; use of benzodiazepines and zolpidem; prescription of drugs for comorbid medical conditions; the use of psychotherapy and neuromodulatory interventions.

### 2.3. Statistical Analysis

We estimated a minimum sample size of 382 referrals/participants, calculated as a representative sample size of the reference area population, using the site http://www.raosoft.com/samplesize.html (accessed on 14 October 2024), for a confidence interval (CI) of 95%. Descriptive statistics were performed for all variables. Univariate inferential statistical tests were used to compare the DTD and non-DTD groups. For continuous outcome variables (age, DM-TRD scores, CCI scores, psychiatric consultations, emergency room visits, and psychiatric hospitalization days), the groups were compared using independent samples t-tests. Effect sizes were estimated based on Cohen’s d values, with d values > 0.5 considered to represent potentially meaningful differences. All other outcome variables were categorical; groups were compared using chi-square tests (χ^2^). Effect sizes for differences in the use of therapeutic interventions were estimated using Cramer’s V statistic, with values around 0.1, 0.3, and 0.5 indicating small, medium, and large effect sizes, respectively. A DM-TRD score raincloud plot was built to explore the differences between DTD and non-DTD groups. Correlation analysis and linear regression were used to explore the associations and analyze the relations between DM-TRD scores, CCI scores, number of psychiatric consultations, emergency room visits, and psychiatric hospitalization days. Pearson correlations heatmaps were made as visually sound options to illustrate correlation patterns. The statistical analysis was performed using JASP version 0.18.3 [24].

### 2.4. Ethics

The study was approved by the Ethics Committee of the FMUC—Faculty of Medicine, University of Coimbra, Portugal (CE-160/2023).

## 3. Results

### 3.1. Patient Selection

**Figure 1:**
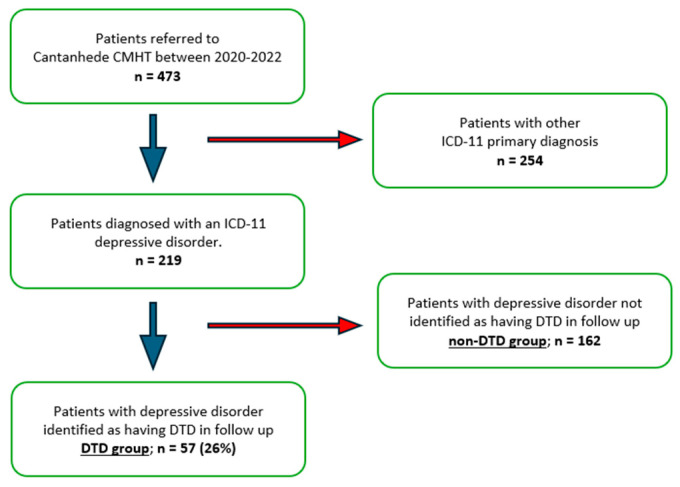
Patient selection flow diagram.

Figure 1 summarizes the patient selection flow diagram. Between 1 January 2020, and 31 December 2022, 473 patients were referred to Cantanhede CMHT for a first assessment consultation, and 219 patients met ICD-11 criteria for a primary diagnosis of any depressive disorder (codes 6A7x) [21]. During follow-up, assistant psychiatrists identified 57 patients with presumed DTD (26%) based on the international consensus statement definition of DTD as “depression that continues to cause significant burden despite usual treatment efforts” [15].

### 3.2. Sociodemographic, Clinical, and Service Use Variables

Table 1 provides a summary of sociodemographic, clinical, and service utilization variables. There were no statistically significant differences between the DTD and non-DTD groups in age and gender. There were no statistically significant differences between the DTD and non-DTD groups in the frequency of psychosocial stressors associated with depressive illness. The DTD group had significantly higher rates of chronic depressive episodes (47.4% vs. 5.6%; *p* < 0.001; V = 0.539) and significant differences in depression severity (*p* = 0.005; V = 0.420). There were statistically significant differences between the DTD and non-DTD groups in service use variables, with more psychiatric consultations (10.26 consultations vs. 6.00 consultations; *p* < 0.001; d = 1.068), emergency room visits (3.68 vs. 0.76; *p* = 0.006; d = 0.766), and psychiatric hospitalization days (1.95 vs. 0.09; *p* < 0.001; d = 0.516) in the DTD group.

### 3.3. Treatment Resistance Variables

**Figure 2:**
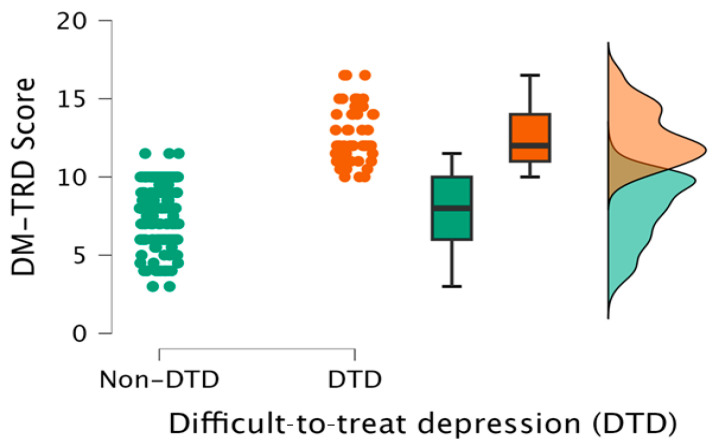
DM-TRD score raincloud plot (DTD group vs. non-DTD group).

The differences between DTD and non-DTD groups in DM-TRD are displayed in Figure 2 and summarized in Table 1. The DM-TRD scores are statistically significant higher in DTD group than non-DTD group, with an effect size that suggests a meaningful difference (12.63 vs. 7.88; *p* < 0.001; d = 2.330).

### 3.4. Psychiatric and Medical Comorbidity

The differences between the DTD and non-DTD groups in psychiatric and medical comorbidities are summarized in Table 2. There were statistically significant differences in the prevalence of comorbid anxiety symptoms (89.5% vs. 57.4%; *p* = 0.011; V = 0.297) and personality disorders and related traits (31.6% vs. 3.8%; *p* < 0.001; V = 0.392), with higher rates in the DTD group. No statistically significant differences between the DTD and non-DTD groups in rates of bodily distress disorder and substance use disorders were found. The DTD group had more severe medical comorbidities, as indicated by statistically significant higher scores in CCI compared to the non-DTD group, with an effect size that suggests a meaningful difference (1.89 vs. 0.81; *p* < 0.001; d = 0.910).

### 3.5. Treatment Interventions for Depression

Table 3 summarizes the differences between the DTD and non-DTD groups related to the implementation of various antidepressant psychopharmacological and psychotherapeutic treatment interventions. There were statistically significant differences in the use of some specific antidepressant drugs, with higher rates of prescription of SNRI (31.6% vs. 9.3%; *p* = 0.019; V = 0.274) and Mirtazapine in the DTD group (52.6% vs. 24.2%; *p* = 0.021; V = 0.270). There were no statistically significant differences between the DTD and non-DTD groups in the use of antidepressant combination strategies. However, there were statistically significant differences in the use of antidepressant augmenting strategies, with higher rates in the DTD group (47.4% vs. 18.5%; *p* = 0.014; V = 0.288). There were no statistically significant differences between the DTD and non-DTD groups in the use of benzodiazepines or zolpidem. There was a trend toward higher use of psychotherapy in the DTD group, but without reaching statistical significance (26.3% vs. 9.3%; *p* = 0.063; V = 0.218). Transcranial magnetic stimulation was used in only one patient from the DTD group, and electroconvulsive therapy was not used in any patient.

### 3.6. Prescription of Drugs for Medical Comorbid Disorders

The differences between the DTD and non-DTD groups in the prescription of some classes of drugs used in the treatment of medical conditions are displayed in Table 4. There were statistically significant differences in the prescription of anticoagulant/antiplatelet drugs (21.0% vs. 1.9%; *p* = 0.004; V = 0.334), opioid analgesics (31.6% vs. 7.4%; *p* = 0.008; V = 0.308), and NSAIDs (42.1% vs. 7.4%; *p* < 0.001; V = 0.411), with higher rates of prescription in the DTD group. There were no statistically significant differences between the DTD and non-DTD groups in the use of statins, antihypertensive drugs, antidiabetic drugs, aspirin, and bronchodilators.

### 3.7. Associations Between Treatment Resistance, Medical Comorbidity, and Service Use

**Figure 3:**
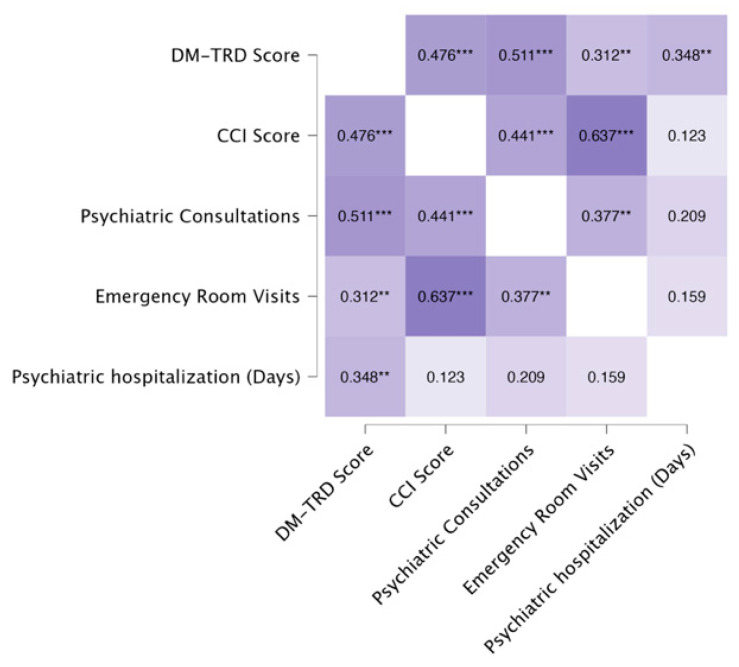
Pearson’s heatmap—correlations between DM-TRD score, CCI score, and service use variables. (* *p* < 0.05; ** *p* < 0.01; *** *p* < 0.001).

**Figure 4:**
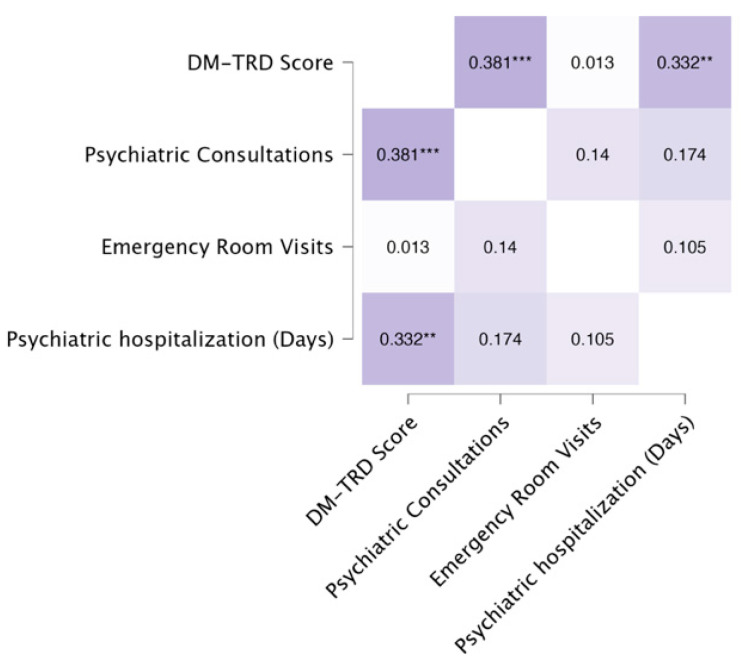
Partial Pearson’s heatmap—partial correlations between DM-TRD score and service use variables, adjusted for CCI scores. (* *p* < 0.05; ** *p* < 0.01; *** *p* < 0.001).

The heatmap in Figure 3 displays the Pearson correlations between DM-TRD scores, CCI scores, and service use variables. There were statistically significant correlations between DM-TRD and CCI scores, between DM-TRD scores and all service use variables (psychiatric consultations, emergency room visits, and psychiatric hospitalization days), and between CCI scores and psychiatric consultations as well as emergency room visits. However, when we performed a partial correlation analysis between DM-TRD scores and service use variables, with CCI as a covariate, only the correlations between DM-TRD scores and psychiatric consultations and between DM-TRD scores and psychiatric hospitalizations remained statistically significant (Figure 4).

**Figure 5:**
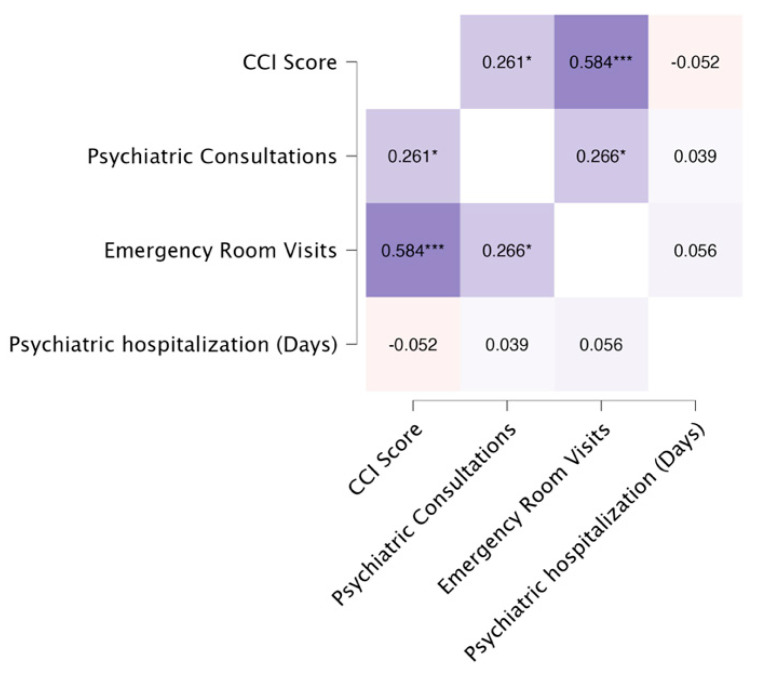
Partial Pearson’s heatmap—partial correlations between CCI score and service use variables, adjusted for DM-TRD score. (* *p* < 0.05; ** *p* < 0.01; *** *p* < 0.001).

On the other hand, when we performed a partial correlation analysis between CCI scores and service use variables, with DM-TRD as a covariate, the correlations between CCI scores and psychiatric consultations and between CCI scores and emergency department visits remained statistically significant (Figure 5).

We performed a linear regression to model the relationship between DM-TRD score, CCI score, and psychiatric consultations, using DM-TRD score and CCI score as predictors and psychiatric consultations as the outcome. Variables selected for the model were those that maintained significant correlation in partial correlation analysis, despite adjustment for the two relevant variables (e.g., variable of interest vs. psychiatric consultations), as a strategy to address multicollinearity. The overall regression was significant: F (2, 216) = 48.753, *p* < 0.001. The model explains about 30% of the variance in psychiatric consultations (Adjusted R^2^ = 0.305). Table 5 provides information about the regression coefficients for the predictors. Depression treatment resistance and medical comorbidities were significant predictors, both showing a positive relationship with the number of psychiatric consultations.

## 4. Discussion

The DTD prevalence in the clinical population studied in our work was approximately 26%. To our knowledge, this is the second epidemiological study focusing on DTD. Given the conceptual flexibility in the DTD definition, intended to be used in a clinical context reflecting the interactions between the patient and the healthcare practitioner and dependent on local treatment guidelines and practices, it is expected that DTD prevalence rates differ between empirical studies conducted in different healthcare settings. In the single previous observational study, conducted in the UK using a natural language processing model to explore differences between DTD and non-DTD groups on disease characteristics, comorbidities, and treatment histories in a sample of 28,184 patients with depression, the prevalence rate of DTD was 19% [20]. In that study, DTD was pragmatically operationalized with the following criteria: the first episode of depression had been coded at least 3 years previously; the patient had a current chronic episode of depression (more than 2 years) or had multiple recurrences (at least three episodes), and there were problems in finding a tolerable and effective treatment as indicated by having at least four antidepressant treatments, of which at least two were drugs for depression [20]. This pragmatic operationalization of the DTD definition was more restrictive than ours. In our study, we asked the community team psychiatrist to identify the patients they judged to meet the criteria of the international consensus definition, with the objective of mimicking the interactive nature of this formulation in a clinical context. The differences in DTD definition operationalization, study methodology, and healthcare contexts between these studies may explain the differences in prevalence.

In terms of clinical variables, it was clear that the DTD group had statistically significant higher rates of chronic depressive episodes and depression severity compared to the non-DTD group. There were also statistically significant differences between the DTD and non-DTD groups in service use variables, with more psychiatric consultations, emergency room visits, and psychiatric hospitalization days in the DTD group. These results are in accordance with available literature [20].

Compared to the non-DTD group, patients with DTD had significantly higher scores on a multidimensional scale that measures treatment resistance in depression (DM-TRD), which was also concluded by other studies [13,20]. This finding suggests that our operationalization of the DTD definition is compatible with a dimensional perspective of depression treatment refractoriness in a real-world clinical context. According to the literature, the DM-TRD has been proven to predict the clinical outcome of patients with depressive disorders. It was also reported that higher scores were associated with a more significant burden during the follow-up of patients with depressive disorder [13], which is compatible with our findings. Our results are also compatible with the view that DTD and TRD are related and somewhat overlapping concepts, but with key differences.

Our study showed that DTD patients had higher rates of comorbid anxiety symptoms, comorbid personality disorders and traits, and higher levels of medical comorbidity. These results are compatible with the findings of other observational real-world studies of DTD patients [20], where the rates of comorbid illness (both mental and physical) are higher in the DTD group compared to the non-DTD group. These higher levels of psychiatric and medical comorbidity can be challenging when treating depressed patients in a real-world healthcare setting, contributing to lower rates of response and remission, higher probability of relapse and recurrence, more difficulty achieving the premorbid level of functioning, lower quality of life, and a higher frequency of treatment side effects and contraindications. All these outcomes can contribute to the clinician’s perception of the difficulties when treating depressed patients with comorbid conditions [15]. Our findings suggest that the mental health service burden is related concurrently with depression treatment refractoriness and medical comorbidity. We found not only significant correlations between DM-TRD and medical comorbidity, as measured by CCI, but also associations between CCI and the frequency of psychiatric consultations and emergency room visits. Medical comorbidity is a variable that is not included in the different conceptualizations of TRD but is an important area to consider in the assessment of DTD [20].

In terms of the antidepressant prescription profile, when comparing DTD vs. non-DTD patients, SNRIs and mirtazapine were more frequently prescribed to the first group. These prescription practices are compatible with depression guidelines recommending the use of specific antidepressants to address common residual depressive symptoms like anxiety, sleep problems, cognitive difficulties, and somatic symptoms [25]. A recent meta-analysis also showed that the use of presynaptic alpha-2 autoreceptor antagonists like mirtazapine in combination with SSRIs or SNRIs had superior treatment outcomes compared to other combinations [26], suggesting that it can constitute a therapeutic option to consider in DTD patients. There was also a significantly higher proportion of antidepressant augmentation strategies in DTD patients compared to non-DTD patients. In a similar UK study [20], it was reported a greater proportion of antidepressant augmentation treatment in DTD patients and a consistent trend for higher dosages. One of the differences between our work and the previously reported secondary care UK study is that we did not observe statistically significant differences in the use of antidepressant combination strategies between DTD and non-DTD patients. In that study, it was clearly demonstrated that the use of intensive combination therapies, including non-pharmacological interventions, was more frequent in the DTD group [20].

In terms of other non-psychiatric drugs, we studied the role of analgesics in DTD and concluded that they were generally more frequently prescribed to DTD patients than non-DTD patients. DTD patients constitute a heterogeneous group, and chronic low-grade inflammation may play a role in the pathophysiology of treatment-resistant depression, at least in a subset of patients [26,27]. The literature also reports a frequent comorbidity between depression and osteoarticular disorders [28], associations between treatment-resistant depression and the risk of autoimmune disorders [27,29], and a positive correlation between both anxious and depressive symptomology and pain severity [30]. All these factors could provide a partial explanation for the more frequent use of analgesic drugs in DTD patients in our sample. The prescription of anticoagulants was also more frequent in DTD patients, despite the literature reporting that depression is an independent risk factor for major bleeding in anticoagulated individuals (particularly in patients with atrial fibrillation) [31,32], highlighting the importance of a comprehensive assessment of medical comorbidities in patients with depressive disorders.

Finally, when comparing the number of psychiatric consultations and emergency room visits, it was evident that DTD patients had a statistically significant higher number compared to non-DTD patients. These results were also expected since the definition of DTD includes patients who are heavier consumers of mental health care services, including consultations, emergency room visits, and psychiatric hospitalizations [20].

Of relevance to the results of our study, its timeframe was influenced by the SARS-CoV-2 pandemic. According to the World Health Organization (WHO), MDD was already the single largest contributor to loss of healthy life, and this contribution has apparently further increased during COVID-19 [8].

Cultural and socioeconomic aspects should be considered, given epidemiological studies show that while Portugal is one of the most affected European countries by depression, care provision has relevant barriers [19]. TRD diagnosis and treatment are usually performed by psychiatrists but limited by lack of access to specialist care and to treatment options like neuromodulation and psychotherapy [19].

Our study has some important limitations. Compared with a previous observational real-world DTD study, our sample is smaller and restricted to a unique community mental health team-treated population, suggesting caution regarding the generalization of our findings to other clinical settings [20]. We are also aware that our operationalization of the DTD definition rests solely on the team psychiatrist’s clinical judgment informed by their knowledge of the DTD literature and not on specified a priori criteria. This was an intentional choice to emulate daily psychiatric practice but differs from the operationalization of the DTD definition in similar studies [20] and did not consider the patient perspective. Another important issue is that the metrics used to characterize the outcome of depression were suboptimal and consisted only of service use variables. To capture longer-term clinical outcomes in DTD, we should consider using integrative metrics that aggregate information over time in symptomatic, functionality, and quality of life domains [32,33]. Retrospective studies have commonly known limitations: when reviewing charts that were originally not designed to collect data for research, some information is bound to be missing, besides selection biases (e.g., of DTD patients) and lost follow ups. In this chart review, EHR available data were insufficient to quantitatively address relevant variables such as symptom severity, functioning and quality of life.

Prospective research designs, using self-report questionnaires to identify probable DTD patients [17], a more comprehensive assessment of psychiatric and medical comorbidities, treatment history and care pathways, and the use of integrative metrics to capture longer-term outcomes in symptomatic and quality of life domains, is a possible avenue for future research projects [33,34].

Epidemiological data about DTD can lead to a broader and more inclusive approach to TRD, highlighting the necessity of considering factors beyond mere resistance to pharmacological interventions. This novel perspective, integrating psychosocial, biological, and interactive dimensions, lays the groundwork for an integrative model of therapeutic management of resistance in depression [14,16].

Increasing awareness among the general public, specialists, and healthcare providers about the significance of these factors in managing DTD can help mitigate the stigma associated with both depression and resistance to treatment, improving patients’ quality of life. Additionally, a deeper understanding of DTD can lead to enhanced patient outcomes [14].

## 5. Conclusions

DTD is a recent conceptualization that extends the TRD model, with important implications for the assessment and management of depressed patients with suboptimal outcomes in a clinical context. It is not a diagnosis per se, but rather a framework or model of care, arguably more appropriate for equating depression in real world clinical practice [20]. To our knowledge, this is the first observational real-world evidence study about DTD in Portugal, and only the second globally, finding a prevalence of 26% of DTD in patients with depressive disorders treated by a Portuguese community mental health team over a 3-year period. Compared with non-DTD depressed patients, the DTD group had higher scores on a multidimensional measure of depression treatment resistance, higher rates of anxiety symptoms and personality disorders and traits, higher scores on a medical comorbidity index, a higher proportion of antidepressant augmentation strategies, and a higher number of psychiatric consultations, emergency room visits, and psychiatric hospitalization days. Treatment resistance and medical comorbidity were independent predictors of the quantity of psychiatric consultations. These results are in accordance with findings from observational real-world evidence studies implemented in other countries, highlighting the importance of adopting a different approach in the assessment and management of patients with depressive disorders that continue to cause a significant burden despite normal treatment efforts. In times where mental health services still require urgent investment and are frequently outpaced by depression’s prevalence, identifying DTD patients and providing them with facilitated and earlier access to available treatment options—including psychotherapy, neurostimulation, and novel pharmacological strategies—is imperative [19].

## Figures and Tables

**Table 1:** Comparison of sociodemographic, clinical, and service use variables between DTD and Non-DTD groups.

Variables		DTD Group (n = 57)	Non-DTD Group (n = 162)	Statistic	*p*-Value	Effect Size
Sociodemographic variables						
Age (years; mean, s.d.)		58.7 (15.1)	57.7 (14.2)	t = −0.278	0.782	*d* = 0.267 ^a^
Gender (women)		84.2%	74.1%	$\chi^{2}$ = 0.808	0.369	*V* = 0.105 ^b^
Clinical variables						
Chronic depressive episodes		47.4%	5.6%	$\chi^{2}$ = 21.178	**<0.001**	*V* = 0.539 ^b^
Depression severity	Subsyndromal	0.0%	27.8%	$\chi^{2}$ = 12.867	**0.005**	*V* = 0.420 ^b^
	Mild	26.3%	37.0%			
	Moderate	57.9%	33.3%			
	Severe	15.8%	1.9%			
Psychosocial stressors		57.9%	57.4%	$\chi^{2}$ = 0.808	0.971	*V* = 0.004 ^b^
DM-TRD Score		12.63 (1.77)	7.88 (2.12)	t = −3.412	**<0.001**	*d* = 2.330 ^a^
Service use variables						
Psychiatric consultations (mean, s.d.)		10.26 (4.99)	6.00 (3.64)	t = −3.968	**<0.001**	*d* = 1.068 ^a^
Emergency room visits (mean, s.d.)		3.68 (7.36)	0.76 (1.21)	t = −2.846	**0.006**	*d* = 0.766 ^a^
Psychiatric hospitalization days (mean, s.d.)		1.95 (6.85)	0.09 (1.10)	t = −3.968	**<0.001**	*d* = 0.516 ^a^

s.d.: standard deviation; DM-TRD: Dutch measure for quantification of Treatment Resistance in Depression; t: independent sample *t*-tests were used to compare groups and ^a^ Cohen’s d values were used to estimate effect size for continuous outcome variables; $\chi^{2}$: chi-square tests were used to compare groups and ^b^ Cramer V statistic was used to estimate effect sizes for categorical outcome variables.

**Table 2:** Comparison of psychiatric and medical comorbidity between DTD and Non-DTD groups.

Variables	DTD Group (n = 57)	Non-DTD Group (n = 162)	Statistic	*p*-Value	Effect Size
Psychiatric comorbidity					
Comorbid anxiety symptoms	89.5%	57.4%	$\chi^{2}$ = 6.418	**0.011**	*V* = 0.297 ^b^
Bodily distress disorder	10.5%	3.7%	$\chi^{2}$ = 1.263	0.261	*V* = 0.132 ^b^
Disorders due to substance use	15.8%	9.3%	$\chi^{2}$ = 0.614	0.433	*V* = 0.092 ^b^
Personality disorders and related traits	31.6%	3.8%	$\chi^{2}$ = 11.192	**<0.001**	*V* = 0.392 ^b^
Medical comorbidity					
CCI score (mean, s.d.)	1.89 (1.66)	0.81 (0.97)	t = −3.412	**<0.001**	*d* = 0.910 ^a^

s.d.: standard deviation; CCI: Charlson Comorbidity Index; t: independent sample t-tests were used to compare groups and ^a^ Cohen’s d values were used to estimate effect size for continuous outcome variables; $\chi^{2}$: chi-square tests were used to compare groups and ^b^ Cramer V statistic was used to estimate effect sizes for categorical outcome variables.

**Table 3:** Comparison of antidepressant treatment (psychopharmacologic and psychotherapeutic interventions) between DTD and Non-DTD groups.

Variables	DTD Group (n = 57)	Non-DTD Group (n = 162)	Statistic	*p*-Value	Effect Size ^a^
Psychopharmacologic intervention					
*SSRI*	94.7%	81.5%	$\chi^{2}$ = 1.930	0.165	*V* = 0.163
SNRI	31.6%	9.3%	$\chi^{2}$ = 5.471	**0.019**	*V* = 0.274
Bupropiom	21.1%	7.4%	$\chi^{2}$ = 2.682	0.102	*V* = 0.102
Mirtazapine	52.6%	24.1%	$\chi^{2}$ = 5.311	**0.021**	*V* = 0.270
Trazodone	36.8%	26.9%	$\chi^{2}$ = 0.817	0.366	*V* = 0.106
Agomelatine	21.0%	7.4%	$\chi^{2}$ = 2.682	0.102	*V* = 0.192
Vortioxetine	10.5%	7.4%	$\chi^{2}$ = 0.181	0.670	*V* = 0.050
Tricyclic antidepressants	21.0%	9.3%	$\chi^{2}$ = 1.809	0.179	*V* = 0.157
AD combination	68.4%	48.1%	$\chi^{2}$ =2.321	0.128	*V* = 0.178
AD augmentation	47.4%	18.5%	$\chi^{2}$ = 6.076	0.014	*V* = 0.288
Benzodiazepines	94.7%	79.6%	$\chi^{2}$ = 2.335	0.126	*V* = 0.179
Zolpidem	15.8%	3.7%	$\chi^{2}$ = 3.218	0.073	*V* = 0.210
Psychotherapeutic intervention					
Psychotherapy	26.3%	9.3%	$\chi^{2}$ = 3.459	0.063	*V* = 0.218

SSRI: Selective Serotonin Reuptake Inhibitors; SNRI: Serotonin and Norepinephrine Reuptake; inhibitors; AD: Antidepressants; $\chi^{2}$: chi-square tests were used to compare groups, and ^a^ Cramer *V* statistic was used to estimate effect sizes for categorical outcome variables.

**Table 4:** Comparison of pharmacological treatments for comorbid medical disorders between DTD and Non-DTD groups.

Variables	DTD Group (n = 57)	Non-DTD Group (n = 162)	Statistic	*p*-Value	Effect Size ^a^
Statins	26.3%	27.8%	$\chi^{2}$ = 0.015	0.902	*V* = 0.014
Antihypertensives	47.4%	37.0%	$\chi^{2}$ = 0.627	0.429	*V* = 0.093
Antidiabetic agents	10.5%	11.1%	$\chi^{2}$ = 0.005	0.944	*V* = 0.008
Anticoagulants	21.0%	1.9%	$\chi^{2}$ = 8.121	**0.004**	*V* = 0.334
Aspirin	15.8%	11.1%	$\chi^{2}$ = 0.285	0.594	*V* = 0.062
Bronchodilators	10.5%	1.9%	$\chi^{2}$ = 2.684	0.101	*V* = 0.192
Analgesics—Opioids	31.6%	7.4%	$\chi^{2}$ = 6.946	**0.008**	*V* = 0.308
Analgesics—NSAID	42.1%	7.4%	$\chi^{2}$ = 12.319	**<0.001**	*V* = 0.411

NSAID: Nonsteroidal anti-inflammatory drug; $\chi^{2}$: chi-square tests were used to compare groups, and ^a^ Cramer *V* statistic was used to estimate effect sizes for categorical outcome variables.

**Table 5:** Regression coefficients—regression analysis of the relationship between depression treatment resistance (DM-TRD score), medical comorbidity (CCI score) and number of psychiatric consultations.

Predictors	B	Std. Error	β	t	*p*-Value
DM-TRD Score	0.589	0.097	0.389	6.055	<0.001
CCI Score	0.888	0.223	0.255	3.974	<0.001

## Data Availability

The data presented in this study are available on request from the corresponding author.
